# Scaling the Variance of a Latent Variable While Assuring Constancy of the Model

**DOI:** 10.3389/fpsyg.2019.00887

**Published:** 2019-04-24

**Authors:** Karl Schweizer, Stefan J. Troche, Christine DiStefano

**Affiliations:** ^1^Institute of Psychology, Goethe University Frankfurt, Frankfurt, Germany; ^2^Department of Psychology, University of Bern, Bern, Switzerland; ^3^Department of Educational Studies, University of South Carolina, Columbia, SC, United States

**Keywords:** scaling, variance parameter, variance of latent variable, confirmatory factor analysis, structural equation modeling, scaling methods, constancy framework

## Abstract

This paper investigates how the major outcome of a confirmatory factor investigation is preserved when scaling the variance of a latent variable by the various scaling methods. A constancy framework, based upon the underlying factor analysis formula that enables scaling by modifying components through scalar multiplication, is described; a proof is included to demonstrate the constancy property of the framework. It provides the basis for a scaling method that enables the comparison of the contribution of different latent variables of the same confirmatory factor model to observed scores, as for example, the contributions of trait and method latent variables. Furthermore, it is shown that available scaling methods are in line with this constancy framework and that the criterion number included in some scaling methods enables modifications. The impact of the number of manifest variables on the scaled variance parameter can be modified and the range of possible values. It enables the adaptation of scaling methods to the requirements of the field of application.

## Introduction

In evaluating the results of factor analysis, the focus is usually on the factor loadings as related to the magnitude and the direction of the relationship to the latent variable. While also a parameter of the model, under factor analysis, the variance of the latent variable is largely ignored as a source of information for evaluation. A reason for ignoring the variance as a source of information is its dependency on the indicator selected for scaling in order to achieve model identification. It is well-known that modifying scaling by replacing one indicator by another one changes the value of the variance among other consequences (e.g., Gonzalez and Griffin, 2001; Steiger, 2002). Such dependency does not endorse the variance of the latent variable as a reliable source of information.

Despite the dependency on indicator selection, factor variance can be an important piece of information for evaluation. Even though it is commonly ignored, the variance of a latent variable has been recognized as a useful source of information for some specific areas, in particular, longitudinal research and invariance analyses (McArdle and Cattell, 1994; Schmitt and Kuljanin, 2008; McArdle, 2009). For example, the variance of a latent variable is used for evaluating development across time and for gaining insight about differences between groups. Besides these statistical approaches, there are further analysis strategies that may profit from comparisons of the variances of latent variables, such as the multitrait-multimethod approach (Marsh and Grayson, 1995) and the bifactor approach (Reise, 2012). Especially when using a multitrait-multimethod design, it may be important to know how large the trait variance is in comparison to the method variance. This information reveals the relative contributions of different measures to the representation of a construct.

The particular interest in the scaling of latent variables has given rise to several specific methods that satisfy the needs of the corresponding areas of research (Little et al., 2006). For example, in longitudinal research (McArdle and Cattell, 1994) it is useful to scale the variance in such a way that it is set equal to one at the first measurement occasion. This approach establishes a baseline, and changes from the baseline to successive measurement occasions are more readily interpretable. Thus, different scaling methods may be of interest to achieve specific goals based upon the design under consideration. However, despite the different goals giving rise to different scaling methods, all methods must be able to preserve the major outcome of a confirmatory factor investigation while scaling transforms a statistic into a new reference system. Therefore, it should be possible to relate the various scaling methods to each other and to integrate them into a common framework.

The available methods for scaling variances (either implicitly or explicitly) include a definition of the relationship between the factor loadings and the variance of the corresponding latent variable (Little et al., 2006). Such a definition is also required in confirmatory factor analysis for specifying the model of the covariance matrix^1^ (Jöreskog, 1970). Therefore, this model is considered as the framework that may preserve the major outcome of an investigation and is suitable for investigating scaling methods. We discuss this point in greater detail in the following sections where different scaling methods are considered and consequences of possible modifications are demonstrated.

## Scaled Variances as Sources of Information

In order to be regarded as an important source of information, the variance of the latent variable must be scaled; i.e., it must be adapted to the reference system of interest. This kind of adaptation requires that a content area is identified that potentially profits from the availability of scaled variances. Some content areas for scaling are already mentioned. In this section the perspectives of models of measurement are used for considering areas that may profit from scaling the variances of latent variables. Furthermore, scaling in confirmatory factor analysis (CFA) is compared with standardization for obtaining meaningful weights in linear regression analysis. Standardized regression weights enable the comparison of the contributions of independent variables to the explanation of the dependent variable.

Before detailing the process, the specificity of the variance characterizing a latent variable needs to be addressed. Both the latent variable and the variance are parts of a tested model and, therefore, to some degree are shaped by the characteristics of this model. The variance of the latent variable is assigned the role of a parameter of a model that is thought to reflect dispersion, but is not equivalent to the variance defined as the sum of squared deviations (Verbeke and Molenberghs, 2003; Stoel et al., 2006). For ease in communication, we stay with the term variance.

At first, the possible advantage of scaling the variance of the latent variable of a one-factor confirmatory factor model is considered. This model relates the *p*×1 vector **x** representing the centered observations to the product composed of the *p*×1 vector **λ** representing the factor loadings and of the latent variable ξ and to the *p*×1 vector **δ** representing the error influences (Graham, 2006):

(1) $$\begin{array}{l} \text{x}=\text{λ}\xi +\text{δ}. \end{array}$$

There is also an extended version of this model (Miller, 1995; Raykov, 1997). It additionally includes the *p*×1 vector **μ** of latent intercepts and applies to the non-centered observations **X** instead of the centered observation **x**:

(2) $$\begin{array}{l} \text{x}=\text{μ}+\text{λ}\xi +\text{δ}. \end{array}$$

This unidimensional model mainly serves the investigation of the structural validity and also of the convergent and discriminant validity of scales. Examinations are expected to provide information on the correctness of this model with respect to the given data. If the information suggests correctness (as shown by acceptable fit), it is retained; otherwise it is rejected. No further information requiring scaling is necessary unless there is a repeated application of the model.

The model of measurement of Equation (2) is designed according to the assumption that there is only one systematic source of responding. It ignores, for example, the well-known impurity problem that was observed in cognitive measures (Jensen, 1982; Miyake et al., 2000). It states that it is virtually impossible to complete many cognitive items without stimulating auxiliary processes besides the intended cognitive process. In other words, it is quite likely that there is at least one other process influencing the responses besides the process reflecting the construct, which is in the focus of the scale. This second process needs to be represented in the model of measurement as another source of responding by an additional component. Enlarging the model of measurement of Equation (2) gives the following:

(3) $$\begin{array}{l} \text{x}=\text{μ}+{\text{λ}}_{\text{first}\;\text{source}}\;\xi {\;}_{\text{first}\;\text{source}}+{\text{λ}}_{\text{second}\;\text{source}}\;\xi {\;}_{\text{second}\;\text{source}}+\text{δ} \end{array}$$

where the labels *first source* and *second source* distinguish as subscripts between the construct source reflecting the intended cognitive process and the other source, the auxiliary process.

In the case of the two-factor confirmatory factor model, it may not be sufficient to know that the model is correct because there are two different sources showing different qualities. In the case of the second source being unrelated to the source captured by the scale, the two sources are a “good” source (related to the construct of interest) and a “bad” source (clouding measurement of the construct) and, therefore, it is at least important to know whether the good one dominates the response, and it is even better to be able to show that the influence of the bad source is a minor influence on the responses. This means that the two latent variables constitute a reference system for scaling.

Distinguishing between good and bad sources is not just an idea but a real problem of substantive research. There are, for example, impure measures of working memory capacity showing this characteristic. We mention one major case of controversy that highlights the importance of quantifying the contributions of the additional sources to responding: there are now a number of studies reporting very high correlations between working memory capacity and intelligence suggesting more or less equivalence of working memory capacity and fluid intelligence. However, there is also good reason for suspecting that measures of working memory capacity do not only tap working memory capacity but also processing speed (Chuderski, 2013, 2015). Using a very large sample in an investigation focused on this issue, it was possible to demonstrate that minimizing the possible influence of processing speed lowered the correlation substantially. That processing speed is a threat to the validity of a measure is not only a problem of cognitive research but also of assessment in general. If there is a time limit in testing, processing speed is likely to contribute to performance (Oshima, 1994). The combination of a time limit in testing and processing speed impairs the validity of measurement (Lu and Sireci, 2007).

A similar situation is noted in linear regression analysis with two or more independent variables. The dependent variable is explained/predicted by the independent variables, and it is of interest to know about the relative contributions of the individual independent variables. These contributions are reflected by the regression weights. For demonstrating the structural similarity with the model of Equation (3), assume the dependent variable Y, the independent variables X_1_ and X_2_, the intercept *b*_0_ and the error *e* (notation according to Osborne, 2017) that relate to each other according to the following equation:

(4) $$\begin{array}{l} \text{Y}=b_{0}+b_{1}{\text{X}}_{1}+b_{2}{\text{X}}_{2}+e \end{array}$$

where *b*_1_ and *b*_2_ are the regression weights. Standardized regression weights signify the contributions of independent variables to the explanation of the dependent variable. These regression weights can be compared. For example, the weights can be used for evaluating contributions of independent variables that, for example, may be considered as variables reflecting good and bad sources.

The confirmatory factor model of Equation (3) includes equations showing a structure similar to Equation (4), as is obvious when using a more detailed way of presenting the vectors:

(5) $$\begin{array}{l} \left[\begin{array}{c} {\text{X}}_{1}\\ {\text{X}}_{2}\\ .\\ .\\ {\text{X}}_{p} \end{array}\right]=\left[\begin{array}{c} \mu_{1}\\ \mu_{2}\\ .\\ .\\ \mu_{p} \end{array}\right]+{\left[\begin{array}{c} \lambda_{1}\\ \lambda_{2}\\ .\\ .\\ \lambda_{p} \end{array}\right]}_{\text{first}\;\text{source}}\times \xi_{\text{first}\;\text{source}}\\ \;+{\left[\begin{array}{c} \lambda_{1}\\ \lambda_{2}\\ .\\ .\\ \lambda_{p} \end{array}\right]}_{\text{second}\;\text{source}}\times \xi_{\text{second}\;\text{source}}+\left[\begin{array}{c} \delta_{1}\\ \delta_{2}\\ .\\ .\\ \delta_{p} \end{array}\right] \end{array}$$

There are factor loadings serving more or less the same purpose as the regression weights in regression analysis (λ_*i*_ instead of *b*_*i*_). Although the estimation methods used in confirmatory factor analysis and linear regression analysis may differ from each other and lead to somewhat differing estimates, factor loadings, and regressions weights show some functional similarity.

However, in confirmatory factor analysis, the two sources that are to be compared with each other show not only one factor loadings, but *p* of them. This means that the factor loadings need to be integrated into one statistic. The variance can be this statistic since factor loadings and the variance of the latent variable depend on each other, as is demonstrated in the next section. The dependency is established by a framework. By means of this framework it becomes possible to relate variances scaled with respect to multiple indicators to the initially mentioned scaling by fixing one indicator (e.g., Gonzalez and Griffin, 2001; Steiger, 2002). Given this framework, it is shown in one of the following sections that it possible to achieve scaled variances, which can serve for comparisons like those by standardized regression weights, by one of the scaling methods.

## Constancy due to Scalar Multiplication

This section addresses the issue of constancy regarding the reproduction of the empirical covariance matrix by the model of the covariance matrix, despite scaling variance parameters. It is argued that constancy despite scaling by means of the various methods is accomplished by means of scalar multiplication. Scalar multiplication denotes the multiplication of a scalar and a matrix. The usefulness of scalar multiplication is detailed below.

Constancy is considered with respect to the model of the covariance matrix (Jöreskog, 1970) that is often symbolized by ****Σ****. This matrix (i.e., model of the covariance matrix) is denoted as the *p* × *p* model-implied covariance matrix for *p* manifest variables (**Σ** ∈ ℜ^*p*×*p*^) and is specified to reproduce the *p* × *p* empirical covariance matrix **S** (**S** ∈ ℜ ^*p*×*p*^). Under CFA, the definition of the model ****Σ****, is given by the following equation:

(6) $$\begin{array}{l} \text{Σ}={\text{ΛΦΛ}}^{\prime }+\text{Θ} \end{array}$$

where ****Σ**** is defined as the sum of ****Λ**Φ**Λ**′** and **Θ**. The product ****Λ**Φ**Λ**′** is composed of the *p* × *q* matrix of factor loadings ****Λ**** (**Λ** ∈ ℜ ^*p*×*q*^) (and its transpose ****Λ**′**) and the *q* × *q* matrix **Φ** (**Φ** ∈ ℜ ^*q*×*q*^) consists of the variances and covariances of *q* latent variables. The second component in the equation is the *p* × *p* diagonal matrix of error components **Θ** (**Θ** ∈ ℜ ^*p*×*p*^), which is linked additively to the first component.

The reasoning regarding constancy concentrates on ****Λ**Φ**Λ**′** since constancy of this part of the model with respect to a specific empirical covariance matrix **S** implies that **Θ** is also constant. Scaling the variance parameters of ****Λ**Φ**Λ**′** in a manner that assures constancy means that the product (as a whole) is constant, although the factor loadings and the variance and covariance parameters may change.

A constancy framework for scaling. Assume the *p* × *q* matrices of factor loadings denoted ****Λ**** and Λ^*^ and the *q* × *q* matrices of the variances and covariances of latent variables denoted as **Φ** and Φ^*^. Constancy in the sense of equality of ****Λ**Φ**Λ****and Λ^*^Φ^*^Λ^*^,

(7) $$\begin{array}{l} {\text{Λ}\Phi \Lambda }^{\prime }={\text{Λ}}^{*}{\text{Φ}}^{*}{{\text{Λ}}^{*}}^{\prime }, \end{array}$$

is given if there is a scaling constant *c* (*c* ∈ ℜ ^>0^) such that

Λ^*^ = *c* ****Λ****

and

Φ^*^ = 1/ *c*^2^ **Φ**.

Scaling is achieved by multiplying both ****Λ**** and **Φ** with *c* respectively the inverse of its square. In the following section it is demonstrated that the available scaling methods can be described in terms of this framework.

In order to ensure that the stated equality is correct, a proof is provided. The proof consists of three parts:

- transformation of the left-hand side of Equation 7 to the right-hand side to illustrate equivalence (Proof 1)
- demonstration that the products of matrices included in Equation 7 produce matrices of the same size (Proof 2), and
- demonstration that all entries of the two products of matrices are the same (Proof 3).

**Proof 1**. First, *c* ∈ ℜ ^>0^ is introduced into the left-hand side of Equation 7:

$$\begin{array}{l} {\text{Λ}\Phi \Lambda }^{\prime }=1\times {\text{Λ}\Phi \Lambda }^{\prime }=\frac{c\times c}{c\times c}\times {\text{Λ}\Phi \Lambda }^{\prime }=c\times \frac{1}{c^{\text{2}}}\times c\times {\text{Λ}\Phi \Lambda }^{\prime } \end{array}$$

The × symbol is used for explicitly emphasizing some cases of multiplication. As *c* is a scalar, **Λ** and **Φ** are matrices and the entries of the matrices are real numbers. Thus, the commutative and associative properties of scalar multiplication enable reordering of the scalars:

$$\begin{array}{l} c\times \frac{1}{c^{\text{2}}}\times c\times \text{Λ}\Phi {\text{Λ}}^{\prime }=c\times \text{Λ}\times \frac{1}{c^{\text{2}}}\times \text{Φ}\times c\times {\text{Λ}}^{\prime }\\ \;=\left(c\times \text{Λ}\right)\times \left(\frac{1}{c^{\text{2}}}\times \text{Φ}\right)\times \left(c\times {\text{Λ}}^{\prime }\right)\\ \;=\left(c\text{Λ}\right)\left(\frac{1}{c^{\text{2}}}\text{Φ}\right)\left(c{\text{Λ}}^{\prime }\right) \end{array}$$

Finally, a product term is achieved that includes components that are in line with the replacement rules introduced in combination with Equation (2), Λ^*^ = *c* ****Λ**** and Φ^*^ = 1/ *c*^2^ **Φ**:

$$\begin{array}{l} \left(c\Lambda \right)\left(\frac{1}{c^{\text{2}}}\Phi \right)\left(c\Lambda^{\prime }\right)={\text{Λ}}^{*}\Phi^{*}\Lambda^{*}\prime . \end{array}$$

**Proof 2**. Since the product of the matrix of factor loadings and of the matrix of variances and covariances (and also the transpose of the matrix of factor loadings) is an additive component of the sum that constitutes the model of the covariance matrix according to Equation 6, the size of **ΛΦΛ**′ is the same as the size of ****Σ**** that is, a *p* × *p* matrix. It remains to demonstrate that Λ^*^Φ^*^Λ^*^′ is also a *p* × *p* matrix. Since *c* is a scalar, it does not change the size of the matrix to which it serves as multiplier. This means that the size of *c* ****Λ**** is the same as the size of ****Λ****, the size of 1/ *c*^2^ **Φ** the same as the size of **Φ**, and the size of *c* ****Λ**′** the same as the size of ****Λ**′**. Consequently, for Λ^*^ = c ****Λ**** and Φ^*^ = 1/ c^2^ **Φ**, the size of Λ^*^Φ^*^Λ^*^′ is the same as the size of **ΛΦΛ′**.

**Proof 3**. This proof requires the demonstration that the entries of **ΛΦΛ′** are the same as the entries of Λ^*^Φ^*^Λ^*^′. Both products of matrices are considered as the true part of a *p* × *p* model-implied covariance matrix (i.e., the summand, excluding error of Equation 6); therefore, the entries of the *i*th row and *j*th column are represented by σ_τ*ij*_ and $\sigma_{\tau \text{ij}}^{*}$ across the two matrices, respectively. Given the research interest in investigating the variance at the latent level, **Φ** is assumed to be a diagonal matrix^2^.

In the case of *q* latent variables and diagonal **Φ**, the true (i.e., population) part of the *i*th row and *j*th column σ_τ*ij*_ is given by:

(8) $$\begin{array}{l} \sigma_{\;\tau \;ij}={\text{λ}}_{i1}\Phi_{11}{\text{λ}}_{j1}+\ldots +{\text{λ}}_{iq}\Phi_{qq}{\text{λ}}_{jq}. \end{array}$$

Analogically, the true part of the entry of the *i*th row and *j*th column σ_ij_^*^ is described by the following equation:

(9) $$\begin{array}{l} \sigma_{\;\tau \;ij}^{*}={\text{λ}}_{i1}^{*}\phi_{11}^{*}{\text{λ}}_{i1}^{*}+\ldots +{\text{λ}}_{iq}^{*}\phi_{qq}^{*}{\text{λ}}_{iq}^{*}. \end{array}$$

The next steps make use of scaling constant *c* as introduced in combination with Equation (7). Since Λ^*^ is set equal to *c*****Λ****, the entry of the *i*th row and *j*th column of Λ^*^ (i.e., λ_ij_^*^) can be replaced by the entry of the *i*th row and *j*th column of *c*****Λ**** (i.e., *c*λ_*ij*_). Furthermore, as Φ^*^ corresponds by definition to 1/*c*^2^ **Φ**, the entry of the *i*th row and *j*th column of Φ^*^ that is ϕ_ij_^*^ can be replaced by the entry of the *i*th row and *j*th column of 1/*c*^2^ **Φ** that is 1/*c*^2^ϕ_*ij*_ such that:

$$\begin{array}{l} \sigma_{\;\tau \;ij}^{\text{*}}=c{\text{λ}}_{i1}\frac{1}{c^{2}}\phi_{11}c{\text{λ}}_{j1}+\ldots +c{\text{λ}}_{iq}\frac{1}{c^{2}}\phi_{qq}c{\text{λ}}_{jq}. \end{array}$$

Because scalar multiplication is also distributive, the equation can be further transformed into:

$$\begin{array}{l} \sigma_{\;\tau \;ij}^{\text{*}}=c\times \frac{1}{c^{2}}\times c\times \left({\text{λ}}_{i1}\phi_{11}{\text{λ}}_{j1}+\ldots +{\text{λ}}_{iq}\phi_{qq}{\text{λ}}_{jq}\right). \end{array}$$

Since the sum given in parentheses of the right-hand side of this equation corresponds to the right-hand side of Equation (8), it can be replaced by the left-hand side of Equation 8:

$$\begin{array}{l} \sigma_{\;\tau \;ij}^{\text{*}}=c\times \frac{1}{c^{2}}\times c\times \sigma_{\;\tau \;ij}. \end{array}$$

In the final step, coefficients are arranged to provide a ratio that amounts to one:

$$\begin{array}{l} \sigma_{\;\tau \;ij}^{\text{*}}=\frac{c^{2}}{c^{2}}\times \sigma_{\;\tau \;ij}=\sigma_{\;\tau \;ij}\;■ \end{array}$$

## The Integration of the Scaling Methods into the Constancy Framework

Given that the proof applies to all *p* × *q* matrices of factor loadings ****Λ****, it also applies to all *p* × 1 matrices of factor loadings, referred to as *p* × 1 vectors of factor loadings (**λ**). In this case, the matrix of variances and covariances, **Φ**, reduces to the scalar, ϕ. This scalar is the *variance parameter* which represents the variance of the latent variable in the model of the covariance matrix. The status of this parameter as variance has been questioned since it can be assigned a negative value in the process of parameter estimation (Verbeke and Molenberghs, 2003; Stoel et al., 2006).

In this case of a *p* × 1 vector of factor loadings, Equation (7) reduces to:

(10) $$\begin{array}{l} \text{λ}\text{ϕ}{\text{λ}}^{\prime }={\text{λ}}^{*}{\text{ϕ}}^{*}{{\text{λ}}^{*}}^{\prime } \end{array}$$

if there is a scaling constant *c* ∈ ℜ ^>0^such that λ^*^ = *c* **λ** and ϕ^*^ = 1/*c*^2^ϕ.

The one-factor version of the constancy framework, as described by Equation (10) in combination with the two replacement rules, provides the basis for the following equation that related the scaled variance parameter ϕ_sc_ to the scaling constant *c* and to the original variance parameter ϕ:

(11) $$\begin{array}{l} \phi_{\text{sc}}=\frac{1}{{\text{c}}^{2}}\phi . \end{array}$$

Scaling the variance parameter through use of Equation (11) is a general scaling method, as *c* may be selected to represent different scaling methods. Furthermore, this equation can be used to investigate the properties of specific scaling methods and to compare their effects. The following subsections relate this approach to available scaling methods, including the marker-variable method, the reference-group method and criterion-based methods (e.g., effect-coding method; Little et al., 2006; Little, 2013). In the following subsections, each method is described.

### The Marker-Variable Method

**Figure 1:**
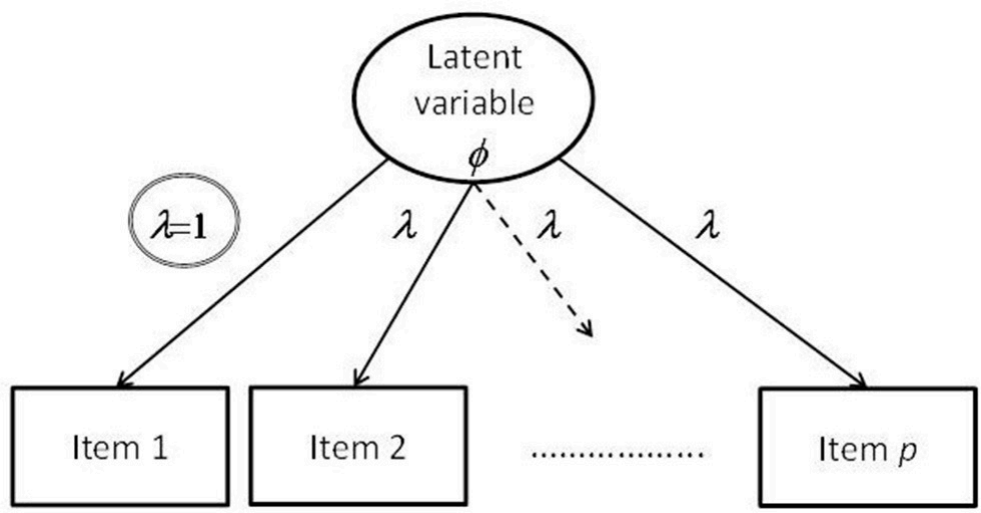
Illustration of a confirmatory factor model with a factor loading constrained according to the marker-variable method.

This frequently used method for scaling the variance parameter states that a value of one is assigned to one of the factor loadings (i.e., a marker variable) while the other factor loadings and the variance parameter of the latent variable are freely estimated. Such a configuration of free and fixed factor loadings is illustrated by Figure 1. A double circle identifies the factor loading selected for serving as indicator.

However, the influence of the marker variable is incorporated into the variance of the latent variable. Integrating this specific method into the constancy framework requires the choice of *c* with respect to the originally selected factor loading λ_*i*_ such that:

(12) $$\begin{array}{l} 1={\text{λ}}_{\text{i}}^{*}=\text{c}{\text{λ}}_{\text{i}} \end{array}$$

where λ_*i*_ refers to the left-hand part of Equation (10) and $\lambda_{i}^{*}$ to the right-hand part. If λ_*i*_ > 0 then *c* ∈ ℜ ^>0^. Given the original variance parameter, ϕ, the scaled variance parameter ϕ_sc_ is obtainable by means of Equation (11).

### The Reference-Group Method

The reference-group method requires that the value of one is assigned to the variance parameter (i.e., standardized latent variables) while all factor loadings are freely estimated. This means that

(13) $$\begin{array}{l} 1=\phi_{\text{sc}}=\frac{1}{{\text{c}}^{2}}\phi . \end{array}$$

**Figure 2:**
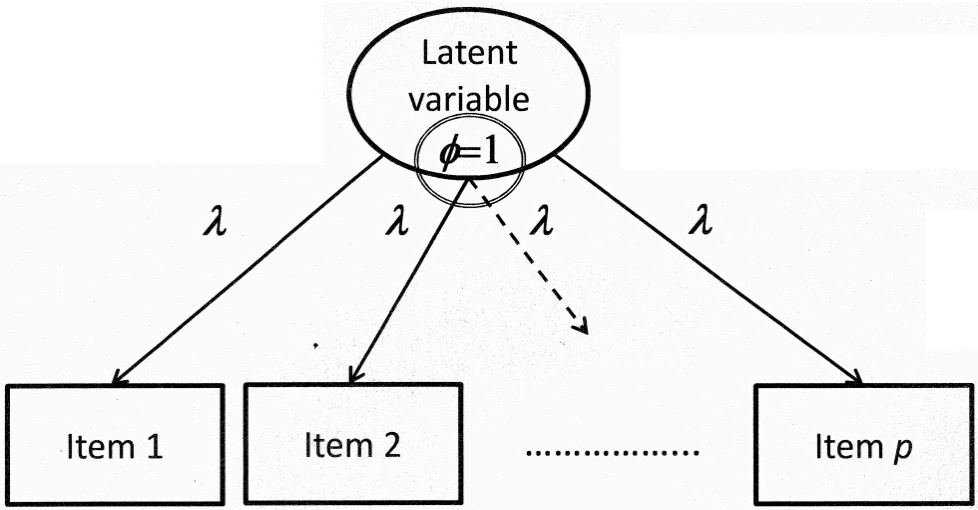
Illustration of a confirmatory factor model with the variance of the latent variable constrained according to the reference-group method.

Figure 2 includes the graphical illustration of major parts of a model of measurement with the variance parameter ϕ set equal to one.

If ϕ_sc_ corresponds to the original variance parameter ϕ, *c* is equal to one. Otherwise, if ϕ is given, *c* is obtainable by means of a reordered version of Equation (11):

$$\begin{array}{l} c=\sqrt{\frac{\phi }{\phi_{\text{sc}}}}. \end{array}$$

### Criterion-Based Methods

Methods including a criterion number, *p*_c_, are referred to as criterion-based methods. The number selected as criterion is related to the sum of factor loadings or the sum of squared factor loadings. Criterion-based methods differ from each other in the number selected as the criterion and the way of summing the factor loadings. First, there is the effect-coding method (Little et al., 2006) that is equivalent to effect-coding used in analysis of variance where factor loadings are replaced by numbers that represent the coding of the effect. These numbers must be adjusted in such a way that their sum equals the number of manifest variables (*p*) and the adjusted numbers are used in the estimation of the variance parameter. In an example provided by Little et al. (2006), each one of the factor loading is set equal to one. It is highlighted that the estimate of the latent variance corresponds to the average of the indicator variables' variances (p. 63).

Equation (14) gives the formal representation of the basic characteristic of this method; that is, the selection of constraints such that the sum corresponds to *p*_c_. In considering the scaling constant, *c*, the method is related to the outlined constancy framework:

(14) $$\begin{array}{l} p_{\text{c}}=1^{\prime }{\text{λ}}_{\text{coding\_constraints}}^{*}=1^{\prime }\text{c}{\text{λ}}_{\text{coding\_constraints}} \end{array}$$

where **1** is a *p* × 1 vector of ones, $\lambda_{\text{coding}\text{\_}\text{constraints}}^{*}$ the vector of adjusted numbers serving as factor loadings and **λ**_coding_constraints_ the vector of original numbers selected for coding the effect. The scaling constant *c* is necessary whenever the numbers selected for coding the effect do not directly sum to *p*_c_.

A second criterion-based method relates the criterion number to the sum of squared factor loadings that was suggested for investigations focusing on variances and covariances (Schweizer, 2011). The number of manifest variables *p* is set equal to the product of the *p* × 1 vectors of adjusted factor loadings λ^*^, respectively the vectors of original factor loadings **λ** with multiplier *c*:

(15) $$\begin{array}{l} p_{\text{c}}=\text{λ}{*}^{\prime }\text{λ}*=c{\text{λ}}^{\prime }c\text{λ}. \end{array}$$

Using principles of scalar multiplication, the *c*s can be put in front of the product of vectors so that:

(16) $$\begin{array}{l} p_{\text{c}}={\text{c}}^{\text{2}}{\text{λ}}^{\prime }\text{λ}. \end{array}$$

The product of vectors reveals that in this case the scaling aims at the variance explained by the factor. Given *p*_c_ and **λ**, *c* is obtainable by means of a reordered version of Equation (16).

**Figure 3:**
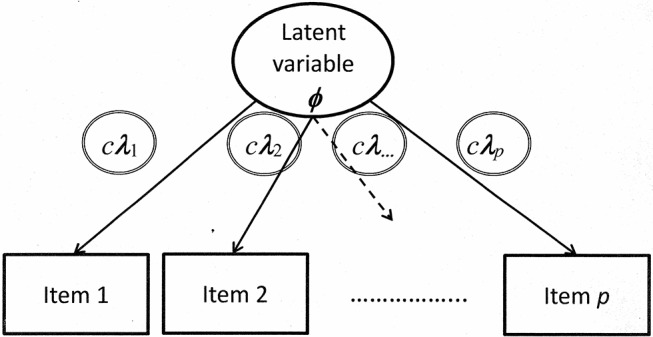
Illustration of a confirmatory factor model with all factor loadings constrained according to the criterion-based methods.

The graphical illustration for demonstrating the criterion-based methods includes products of the scaling constant *c* and λ (see Figure 3).

If λ originates from parameter estimation and not from effect coding, it may be necessary to estimate the value in the first step and fix it for scaling in the second step.

## The Effect of the Criterion Number on the Outcome of Scaling

While the marker-variable method and the reference-group method are rather restricted, the criterion-based methods include a criterion number that enables the adjustment to special expectations regarding the size of scaled variance parameters. This adjustment does not violate the constancy property. Although this criterion number is set equal to the number of manifest variables for good reasons in the version provided by Little et al. (2006), the number is changeable and may be changed to achieving variance values that vary within a smaller or larger range of possible values for the scaled variance parameter.

To demonstrate the effect of different choices of *p*_c_, let *p*_cA_ and *p*_cB_ (where *p*_cA_ > *p*_cB_) be two criterion numbers selected for the scaling of the variance parameter. Given the product **λλ′** and the initial inequity of *p*_cA_ and *p*_cB_, Equation 16 suggests that

$$\begin{array}{l} c_{\text{A}}^{\text{2}}{\text{λ}}^{\prime }\text{λ}>c_{\text{B}}^{\text{2}}{\text{λ}}^{\prime }\text{λ}. \end{array}$$

Because both sides of the inequity include the product **λλ′**, the inequity can be reduced to

$$\begin{array}{l} c_{\text{A}}^{\text{2}}>c_{\text{B}}^{\text{2}}. \end{array}$$

The consequence of this inequity for the scaled variance parameters ϕ_scA_ and ϕ_scB_ when computed according Equation (11) is described by the next inequity:

(17) $$\begin{array}{l} \phi_{\text{scA}}=\frac{1}{c_{\text{A}}^{\text{2}}}\phi <\phi_{\text{scB}}=\frac{1}{c_{\text{B}}^{\text{2}}}\phi \end{array}$$

The scaled variance parameter ϕ_scA_ is smaller than the scaled variance parameter ϕ_scB_ since ϕ_scA_ includes the larger scaling constant *c* as divisor. This inequity reveals that the larger *p*_c_, the smaller the scaled variance parameter.

To demonstrate the practical consequences of selecting different values for *p*_c_, the empirical consequences of changing *p*_c_ are reported in the following section for a number of different conditions. The computations are conducted according to Equations (11, 15). The outset is given by setting the original variance parameter equal to one and the factor loadings to 0.2, 0.4, or 0.6. Furthermore, the number of manifest variables is set to 4, 8, or 12. In the first step, it is investigated how *p*_c_ as proportion of *p*, that is defined to correspond to the number of manifest variables, influences the size of the scaled variance parameter. Three proportions are considered: 1, 1/2, and 1/4. The proportion of 1 requires the consideration of *p*_c_s of 4, 8 and 12, the proportion of 1/2 *p*_c_s of 2, 4, and 6, and the proportion of 1/4 *p*_c_s of 1, 2, and 3.

The results are reported in Table 1. The first to third rows give the results for the original size of the criterion number, the fourth to sixth rows for the half of the original size and the seventh to ninth rows for the quarter of the original size. The inspection of the individual sections of Table 1 reveals that the number of manifest variables has no influence on the size of the scaled variance parameter, whereas the increase of factor loadings leads to an increase of the scaled variance parameter. The results suggest that the larger the factor loadings, the larger the scaled variance parameter. In contrast, the comparison of the sections shows that the smaller the proportion of *p*_c_, the larger the scaled variance parameter. This increase is predicted by the inequity of Equation (17). In the smallest proportion the factor loadings of 0.6 even lead to scaled variance parameters larger than one.

**Table 1:** Sizes of scaled variance parameters for criterion numbers set equal to the number of manifest variables (*p*) or proportions of it (*r*) in combination with different sizes of the factor loadings and numbers of manifest variables.

**Proportion of *r***	**Number of manifest variables *p***	**Sizes of scaled variances**		
		**Factor loading of 0.2**	**Factor loading of 0.4**	**Factor loading of 0.6**
1/1	4	0.04	0.16	0.36
1/1	8	0.04	0.16	0.36
1/1	12	0.04	0.16	0.36
1/2	4	0.08	0.32	0.72
1/2	8	0.08	0.32	0.72
1/2	12	0.08	0.32	0.72
1/4	4	0.16	0.64	1.44
1/4	8	0.16	0.64	1.44
1/4	12	0.16	0.64	1.44

Furthermore, there is the opportunity to define the criterion number *p*_c_ independent of the number of manifest variables. In order to explore this possibility, the criterion number is set equal to 1, 5 and 10. Additionally the numbers of manifest variables (4, 8, 12) and the sizes of factor loadings (0.2, 0.4, 0.6) are varied.

The results are reported in Table 2. This table shows the same structure as Table 1. The comparisons of the three sections display an overall decrease of the scaled variance parameter from the first to the last one. This decrease is in line with the inequity of Equation (17). Furthermore, within the sections there is an increase of the scaled variance parameter from four manifest variables to 12 manifest variables. As also observed in Table 1, there is an increase of the scaled variance parameter associated with the increase of factor loadings.

**Table 2:** Sizes of scaled variance parameters for criterion numbers (*p*_*c*_) independent of the number of manifest variables combined with different sizes of the factor loadings and numbers of manifest variables.

**Criterion number**	**Number of manifest variables**	**Sizes of scaled variances**		
		**Factor loading of 0.2**	**Factor loading of 0.4**	**Factor loading of 0.6**
1	4	0.160	0.640	1.440
1	8	0.320	1.280	2.880
1	12	0.480	1.920	4.320
5	4	0.032	0.128	0.288
5	8	0.064	0.256	0.576
5	12	0.096	0.384	0.864
10	4	0.016	0.064	0.144
10	8	0.032	0.128	0.288
10	12	0.048	0.192	0.432

Taken together, the results show that the increase of the factor loadings leads to an increase of the scaled variance parameter and that an increase of the criterion number leads to a decrease of the scaled variance parameter. Furthermore, the comparison of the results of Tables 1, 2 reveals that linking the criterion number to the number of manifest variables leads to constancy of the scaled variance parameter whereas otherwise, (i.e., when there is independency of the number of manifest variables) an increase of the number of manifest variables leads to an increase of the scaled variance parameter.

## Scaling for Achieving Variances for Comparisons

The achievement of scaled variances for comparing the influences of latent variables on responding like standardized regressions weights in regressions analysis is presented as a major aim in the second section of the paper. For reaching this aim we resort to a basic method of factor analysis for estimating the variance explained by a factor. This method suggests the computation of the sum of squared factor loading **λ′λ**. It can alternatively be achieved by the trace of the corresponding matrix:

$$\begin{array}{l} {\text{λ}}^{\prime }\text{λ}=\text{trace}\left(\text{λ}{\text{λ}}^{\prime }\right). \end{array}$$

Although a variance parameter is not considered, it can be assumed being set equal to one (ϕ = 1) and being omitted for convenience. In order to achieve similarity of the right-hand part of this Equation and the left-hand part of Equation (10) and also Equation (7), ϕ (= 1) is inserted in the right-hand part of this Equation:

(18) $$\begin{array}{l} {\text{λ}}^{\prime }\text{λ}=\text{trace}\left(\text{λ}\phi {\text{λ}}^{\prime }\right). \end{array}$$

In the next step the matrix included in the parentheses is transformed by making use of the second criterion-based method (Equation 15). The criterion number *p*_c_ is set to 1:

(19) $$\begin{array}{l} 1=\text{λ}{*}^{\prime }\text{λ}*=c{\text{λ}}^{\prime }c\text{λ}. \end{array}$$

The scaling framework of Equation (10) respectively Equation (7) enables the replacement of the vectors in the parentheses of Equation (18) and the assignment of the scaling constant as numerator to the variance parameter:

$$\begin{array}{l} {\text{λ}}^{\prime }\text{λ}=\text{trace}\left(\text{λ}*\frac{\phi }{c^{2}}\text{λ}{*}^{\prime }\right) \end{array}$$

Since the ratio of ϕ and *c*^2^ is a scalar, it can be removed from the parentheses and is replaced by the scaled variance parameter ϕ^*^:

$$\begin{array}{l} {\text{λ}}^{\prime }\text{λ}=\phi *\text{trace}\left(\text{λ}*\text{λ}{*}^{\prime }\right). \end{array}$$

Because of setting the criterion number *p*_c_ to 1, the trace must be 1 so that

$$\begin{array}{l} {\text{λ}}^{\prime }\text{λ}=\phi *\times 1=\phi^{*}. \end{array}$$

The contributions of all factor loadings are transferred to the scaled variance parameter. If this method is applied to the variances of two latent variables of the same model, as for example to the latent variables of Equation (3), there are two scaled variances that incorporate the contributions of all factor loadings on the corresponding latent variables. It enables the comparison of the influences of these latent variables on responding.

## Example: Scaling Trait and Method Latent Variables with MTMM

We demonstrate consequences of employing different criterion numbers for scaling the variance of the latent variable through an investigation of a Multitrait-Multimethod (MTMM) design. For illustration, the MTMM matrix from the classic article by Campbell and Fiske (1959) was used; however, we recognize that the original matrix was a synthetic example, and thus, may not demonstrate optimal fit. Using the original MTMM matrix as correlation matrix input for CFA and specifying the model of measurement according to the correlated trait-correlated method model (Widaman, 1985) revealed two problems: (1) two negative error variances and (2) relationships among standardized error variances did not reflect expected relationships for the complements of reliability estimates provided along the main diagonal (0.89, 0.89, 0.76, 0.93, 0.94, 0.84, 0.94, 0.92, 0.85). In order to assure positive values of the error variances and to establish the expected relationship, the main diagonal of the matrix was changed from (1, 1, 1, 1, 1, 1, 1, 1, 1) to (1.145, 1.140, 1.145, 1.005, 0.965, 0.965, 0.940, 1.010, 0.980). Following the argument in justifying the use of the ridge option (Yuan et al., 2011), it was assumed the modification would affect error components of variances but not the systematic components themselves.

Furthermore, the insignificant correlations among the trait and method latent variables were eliminated from the full correlated trait-correlated method model. Only the correlations of the second and third method latent variables (*r* = 0.52) and the first and second trait latent variables (*r* = 0.31) remained. The revised correlated trait-correlated method model yielded good model fit, χ^2^(16) = 17.63, normed χ^2^ = 1.10, RMSEA = 0.014, SRMR = 0.065, CFI = 1.00, GFI = 0.99. This model estimated factor loadings, while the variance parameters of the model were set equal to one for identification.

Various methods for scaling are investigated^3^. At first, the results of criterion-based scaling are reported. Since Equation (14) was proposed for coding effects, Equation (15) guided the computation. Setting the criterion number to 3, that is, to the number of manifest variables for each construct and method led to the following variance parameter estimates: ϕ_method__1_ = 0.286; ϕ_method__2_ = 0.528; ϕ_method__3_ = 0.527; ϕ_trait__1_ = 0.472; ϕ_trait__2_ = 0.481; ϕ_trait__3_ = 0.359. No reported variance estimate was larger than one.

After setting the criterion number to 1, the following estimates of the variance parameter were observed: ϕ_method__1_ = 0.859; ϕ_method__2_ = 1.585; ϕ_method__3_ = 1.582; ϕ_trait__1_ = 1.415; ϕ_trait__2_ = 1.444; ϕ_trait__3_ = 1.076. All estimates of the variances of the trait latent variables were larger than one and two method latent variable variances were larger than one. While not reported, the *t* values for parameter significance testing were independent of the criterion number.

Next, the marker-variable method was used. One of the three factor loadings on each one of these latent variables was set equal to one whereas the remaining factor loadings and the variance parameter were free for estimation. Setting the first factor loading on each factor to one led to the following estimates of the variance parameter: ϕ_method__1_ = 0.291; ϕ_method__2_ = 0.534; ϕ_method__3_ = 0.500; ϕ_trait__1_, = 0.711; ϕ_trait__2_ = 0.745; ϕ_trait__3_ = 0.517. Results for setting the second factor loading on each factor to one were: ϕ_method__1_ = 0.245; ϕ_method__2_ = 0.522; ϕ_method__3_ = 0.543; ϕ_trait__1_ = 0.361; ϕ_trait__2_ = 0.348; ϕ_trait__3_ = 0.299, respectively. Finally, the selection of the third factor loading on each latent variable for constraining values to one provided the following estimates: ϕ_method__1_ = 0.322; ϕ_method__2_ = 0.529; ϕ_method__3_ = 0.539; ϕ_trait__1_ = 0.342; ϕ_trait__2_ = 0.351; ϕ_trait__3_ = 0.260. In sum, different selections led to different estimates of the variance parameters. For example, selecting the first and second manifest variables as markers revealed the variance of the first method latent variable as the smallest one whereas in selecting the third manifest variables as marker the variance of the third trait latent variable was smallest.

The criterion-based method and the marker-variable method were considered for scaling the variance parameters obtained for Campbell and Fiske's MTMM. Different properties of these methods became apparent. The largest estimates were observed for the criterion-based method when the criterion number was one. Setting the criterion number to three led to overall smaller estimates. The marker-variable method led to different rank-orders of the variance estimates for different selections of marker variables. A unique set of variance estimates was not obtainable by means of this method. The reference-group method was not considered since this method only makes sense if dependences among the latent variables can be assumed as in longitudinal research. In contrast, trait and method latent variables are independent of each other.

## Discussion and Conclusions

Although a variance parameter is a necessary component of factor analysis models, researchers often do not consider the effect that the scaling of this parameter has on the variance of the latent variable. One major issue addressed in this paper is the preservation of information when changing from one reference system to another through scaling. Scaling the variance of a latent variable must preserve the result regarding the structure of the data while simultaneously improving interpretability and comparability of the result. The consistency framework presented in this paper reveals how the preservation occurs, and we provide insight into the crucial role of scalar multiplication. Scalar multiplication enables the change of parts of the model of the covariance matrix that is basic to the confirmatory investigation while exhibiting constancy of the product of these parts.

The investigation of the available methods for scaling the variance parameter reveals that the available methods fit to the constancy framework; however, methods present different degrees of flexibility. Whereas, the reference group method is totally fixed, the marker-variable method allows some adaptability to the data in that the method enables the selection of the indicator variable from the set of all manifest variables. We understand that different methods of setting a marker variable for identification may lead to different standard error terms for parameters, and subsequently, different significance test (i.e., Z) values (Gonzalez and Griffin, 2001), the method of scaling latent variables is consistent in terms of fit and parameter estimates.

Criterion-based methods, however, are potentially adaptable to specific needs as the criterion number may be changed to meet a specific situation. The use of a criterion number provides the opportunity to design the method for scaling the variance parameter in such a way that it is possible to: (1) choose between dependency and independency on the number of manifest variables, and (2) opt for lower or larger values of the variance parameter, i.e. different ranges of the possible sizes of the scaled variance parameter, starting with zero.

The application of the scaling methods concentrated on the MTMM provided by Campbell and Fiske (1959). All scaling methods were considered; however, not all of them were able to fit the MTMM matrix. The reference-group method does not apply if there is only one sample; however, it provides a starting point for scaling according to other methods as estimates of the factor loadings are obtained by setting the variances of the latent variables equal to one. The application of the marker-variable methods requires the selection of marker variables; results revealed that different marker-variables lead to different values as the result of scaling. This is not a good property if unique statistics (e.g., means, standard deviations) are expected. Uniqueness of scaled variance estimates are achieved by the criterion-based method.

The criterion-based method also provides an opportunity to achieve scaled variances similar to eigenvalues. Using positive integers as criterion numbers, the largest scaled variance parameters are obtainable for one as criterion number. According to the results of an empirical study, use of the value of one as a criterion number leads to estimates of the variance parameter that correspond to eigenvalues if the model for investigating the data is unidimensional and specific procedural properties are considered (Schweizer et al., 2017). This property enlarges the range of possible applications of scaled variances. Whereas, variance parameters scaled in another way can only be compared with each other, the scaling in using one as criterion number additionally enables comparisons of scaled variances with eigenvalues.

## Author Contributions

KS conceptualized the study and contributed to the writing. ST and CD contribute substantially to the writing of the whole article.

### Conflict of Interest Statement

The authors declare that the research was conducted in the absence of any commercial or financial relationships that could be construed as a potential conflict of interest.

## Footnotes

^1^The model of the covariance matrix refers to the form of the Σ matrix of relationships included in the general factor analysis formula, typically written as: **Σ** = **ΛΦΛ‘+****⊖** and defined in Equation (6).

^2^In this investigation, we are assuming orthogonal factors to place the focus on the decomposition of the latent variance in non-overlapping parts. This assumption is in line with the majority of models of measurement employed in assessment research (Graham, 2006). The omission of the interaction term helps to keep the illustration succinct and of manageable size.

^3^All *t* values for parameter significance were large, with *p* < 0.05 for all tests. We have eliminated *t* values to keep the focus on the variance estimates; however, *t* values are available upon request.
